# Serum magnesium in recovering acute renal failure

**DOI:** 10.4103/0971-4065.43688

**Published:** 2008-07

**Authors:** R. Satish, G. Gokulnath

**Affiliations:** Department of Nephrology, St John's Medical College Hospital, Sarjapur Road, Bangalore - 560 034, India

**Keywords:** Acute renal failure, hypomagnesemia, hypocalcemia, hypokalemia

## Abstract

We studied the manifestations of hypomagnesemia in 50 patients with acute renal failure who had been admitted in our hospital over a period of ten months. All patients with serum creatine ≥ 2 mg/dL and normal baseline levels of serum calcium, magnesium, and potassium as well as normal ECG were included in the study. Patients with multi-organ failure, drug-induced acute renal failure, obstructive uropathy, and alcohol addiction were excluded. The mean age of our study population was 40 ± 15 years, 37 of the patients were male and 13 were female. Hypomagnesemia was observed in 31 patients out of 50 during the recovery period of acute renal failure with symptomatic hypomagnesemia being seen in 23 patients. Serum magnesium levels on the day of admission and during the recovery phase were 2.11 ± 0.38 mg/dL and 1.64 ± 0.41 mg/dL respectively. Paresthesia, irritability, agitation, dysartharia, vertigo, and associated hypokalemia and hypocalcemia were noted in symptomatic hypomagnesemic patients. Treatment of hypomagnesaemia and hypokalemia ameliorated the symptoms. We conclude that these abnormalities produce clinically significant manifestations in recovery phase of acute renal failure and clinicians should pay attention to these.

## Introduction

Acute renal failure (ARF), a syndrome with multiple etiologies, affects approximately 5.7% of all hospitalized patients.^1^,^2^ Approximately 7.2% of all general ward patients and 15% ofall ICU patients develop ARF.^4^ According to Linao *et al.*, the incidence of ARF approaches 200 cases/million adult patients.^3^ ARF has three distinct phases: 1) initial phase, 2) maintenance phase, and 3) recovery phase. ARF is associated with significant morbidity and mortality because of the serious nature of the underlying illness and the high incidence of complications.^4^ Complications include metabolic, cardiovascular, gastrointestinal, neurological, and hematological infections. Electrolyte disturbances in the form of hypocalcemia, hypokalemia, hypophosphatemia, and hypomagnesemia are commonly seen in recovery phase of ARF.^5^

Although magnesium deficiency is a common clinical problem, serum magnesium levels are overlooked in recovering ARF cases. Manifestations of hypomagnesemia are reported to be similar to those of hypokalemia and hypocalcemia, which generally coexist.^1^–^12^ The overall reported prevalence of hypomagnesemia in hospitalized patients is variable and ranges from 4.6–47%, reflecting the type of the patient population studied and different cut-off levels used to define low serum magnesium in these studies.^10^–^12^ Hypomagnesemia produces diverse neuromuscular manifestations such as myclonic jerks, paresthesia, dysartharia, and neuropsychiatric manifestations such as agitation, anxiety, and depression. It also produces ventricular arrythmias and electrolyte abnormalities.^13^ We therefore studied the incidence and manifestations of hypomagnesemia in recovering ARF patients.

## Patients and Methods

This study was a prospective study carried out at a tertiary care center from 01/09/2004 to 30/06/2005. Fifty hospitalized ARF patients were included in the study after obtaining their informed consent. Patients with ARF with serum creatinine >2 mg/dL and normal baseline levels of serum calcium, magnesium, and potassium as well as normal ECG on admission were included in this study. Patients with multi-organ failure and/or ventilatory support, drug-induced or obstructive ARF as well as diabetes and alcohol addiction were excluded. We also excluded patients with serum albumin levels < 3 mg/dL. All these patients were followed till recovery. Serum magnesium, calcium, and potassium levels were estimated in all patients at admission and during the recovery phase of ARF. We defined hypomagnesemia as serum Mg levels < 1.7 mg/dL and symptomatic hypomagnesemia as the presence of hypokalemia/ hypocalcemia / parasthesia vertigo and any ECG changes characteristic of hypomagnesemia. During the recovery phase of ARF, serum magnesium levels were estimated every third day until complete recovery. Those who became hypomagnesemic were evaluated daily for manifestations of hypomagnesemia. In patients with oliguria, the recovery phase of ARF was defined as the increase in urine output of >400 mL/day. In patients who were nonoliguric, the start of a downward trend of blood urea and serum creatinine levels was considered as the first day of recovery. We used a modification of the methyl thymol blue (MTB) complexometric procedure for estimating serum magnesium levels. Data were analyzed using SPSS version 10.0.

## Results

**Fig. 1:**
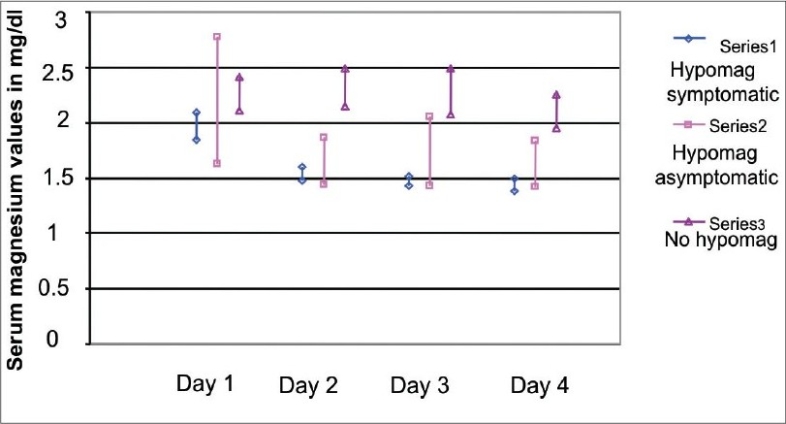
Serum Magnesium levels at different time points

**Fig. 2:**
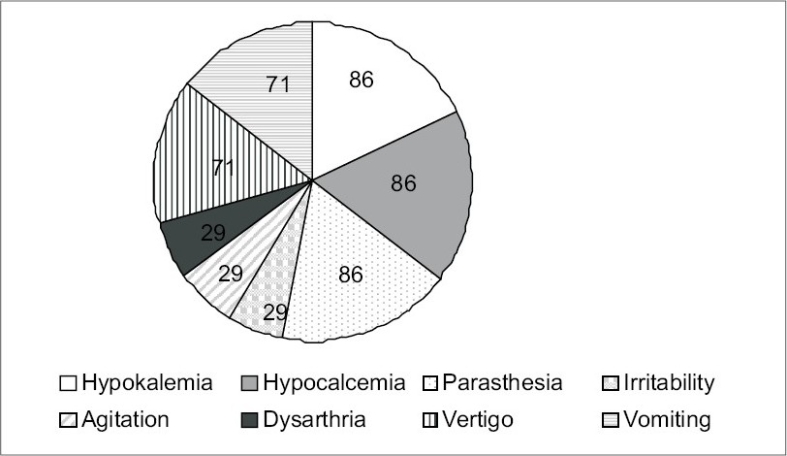
Frequency of common manifestations of hypomagnesemia

Of the 50 patients included in our study, 37 were males and 13 were females with a mean age of 40 ± 15 years [Table 1]. Causes of ARF among the 50 patients in our study were as follows: Sepsis in 30 patients, hypovolemia in 13 patients, septic shock in five patients, and glomerular disease in two patients. Thirty-six of these patients underwent hemodialysis for varying durations and the average hospital stay was 7.74 ± 2.49 days. Thiry-four patients were oliguric and 16 were nonoliguric. All patients had complete recovery. Serum magnesium values on admission and on days 0, 3, and 6 of the recovery phase are depicted in Table 2 and Fig. 1. Out of 50 patients, 31 patients developed hypomagnesemia during the recovery phase of ARF and 23 out of these 31 patients were symptomatic. Twenty-one patients had oliguria and ten patients who were nonoliguric, developed hypomagnesemia. However, the presence or absence of oliguria was not associated with hypomagnesemia during the recovery phase (*P* = 0.2).Clinical manifestations of hypomagnesemia were paresthesia (85.71%), vertigo (71.42%), vomiting (71.42%), irritability (28.57%), agitation (28.57%), dysarthia (28.57%), hypokalemia (85.71%), and hypocalcemia (85.71%) [Fig. 2].

**Table 1:** Demographic and clinical profile of patients (*n* = 50)

Age± SD(in years)	40 ± 15
Sex (M:F)	37:13
Duration of stay in hospital	7.74 ± 2.49 days
Urine output at admission	Oliguric-34, nonoliguric-16
No. of patients who underwent dialysis	36
Mean ± SD dialysis sessions	2.06 ± 1.37
Outcome of acute renal failure	Total recovery in 50 patients

**Table 2:** Serum magnesium values on admission and during recovery phase of acute renal failure

	Serum magnesium (mg/dL)			
	
Hypomagnesemia	On admission	On day 1	On day 3	On day 6
Present (*n* = 31)	2.03 ± 0.41	1.59 ± 0.23	1.53 ± 0.23	1.65 ± 0.36
Absent (*n* = 19)	2.26 ± 0.29	2.32 ± 0.35	2.28 ± 0.44	2.10 ± 0.32

## Discussion

In this study we observed hypomagnesemia in 62% of the patients (31/50) with recovering ARF, 74% of whom were symptomatic. A common cause of hypomagnesemia is the loss of magnesium from the gastrointestinal tract or the kidney. Urinary magnesium loss is often the basis for magnesium depletion, either because of sodium reabsorption in the same tubular segments (magnesium transport passively follows that of sodium), or because of a primary defect in renal tubular magnesium absorption.^14^

Thiazides and loop diuretics inhibit Mg reabsorption but any resulting hypomagnesemia is usually mild because of the increased proximal tubular reabsorption of Mg induced by volume depletion. Renal Mg reabsorption is related to urine flow; hence, long-term parenteral fluid therapy and volume expansion could result in magnesium deficiency. Hence, hypomagnesemia is common in ICU patients.^15^ Hypercalemia and hypocalciuria decrease renal Mg reabsorption, hence, magnesium wasting may be observed in hypercalcemic states such as hyperparathyroidism or malignancy. Diabetes mellitus is the most common cause of hypomagnesemia; probably secondary to glycosuria and osmotic diuresis. Hence, we have excluded diabetes and ICU patients on parenteral therapy from our sudy.

Of the drugs implicated in hypomagnesemia, alcohol is very common, hypomagnesemia being found in 30% of alcoholic patients admitted to the hospital.^16^ Other nephrotoxic drugs include aminoglycoside antibiotics, amphotericin B, cisplatin, cyclosporine, foscarnet, and pentamidine. Hypomagnesemia can persist for a long time after acute tubular damage has been reversed. We therefore excluded alcoholics and drug-induced ARF from our study and none of our patients received diuretics at any time.

Two conditions are associated with primary renal tubular Mg wasting: one is characterized by hypocalcemia, nephrocalcinosis, and a tubular acidification defect, the other, Gitelman's syndrome, is associated with hypercalciuria and a gene encoding for the thiazide-sensitive Na^+^/Ca^2+^ cotransporter.^15^ None of our patients had the above problems.

Hypomagnesemia may also accompany other disorders, including phosphate depletion, hungry bone syndrome after parathyroidectmy and correction of chronic systemic acidosis, postobstructive diuresis, renal transplantation, and the diuretic phase of acute tubular necrosis. The greatest incidence of hypomagnesemia in our study was seen in the diuretic phase of ARF when most patients were off dialysis. Only two of 31 hypomagnesemic patients required dialysis in two sittings of three hours during the recovery phase, which would not have caused hypomagnesemia. There was no association between urine output and symptomatic hypomagnesaemia in our study.

Most of the symptoms of moderate to severe hypomagnesemia [Table 1] are nonspecific and symptomatic magnesium depletion is usually associated with additional ion abnormalities such as hypocalcemia, hypokalemia, and metabolic alkalosis.^17^ In our study, symptoms of hypomagnesemia were seen when serum magnesium levels were below 1.5 mg/dL. Arterial blood gas estimation in 8/31 patients during the recovery phase of ARF did not show any metabolic alkolosis. Hypocalcemia and hypokalemia were seen in our study when serum Mg level were below 1.5 mg/dL. The common symtomatology in our study included parasthesia, irritability, and dysarthria. All above symptoms improved with the correction of hypomagnesemia. Out of 23 symptomatic patients, 15 required intravenous correction: 4 g magnesium sulphate loading, followed by 1 g every six hours. This was given until symptoms improved or serum Mg levels normalized In our study, both hypocalcemia and hypokalemia occurred in 87.71% of the inpatients with serum Mg <1.5 mg/dL.^15^

Magnesium depletion has been associated with acute electrocardiograph changes such as widening of the QRS complex and the appearance of peak T waves. In severe depletion, the PR interval is prolonged with progressive widening of the QRS complex, T-wave inversion, and the appearance of U waves. None of our patients showed any electrocardiological abnormalities.

In conclusion, hypokalemia and hypocalcemia were commonly seen with hypomagnesemia in recovering ARF patients in our study. Treating hypomagnesemia and associated electrolyte abnormalities ameliorated the symptoms. Our findings highlight the need for a large-scale study, including larger numbers of patients and close monitoring for symptoms of hypomagnesemia. We need to rule out other causes which produce similar symptoms and determine whether correction of serum magnesium levels in recovering ARF patients will benefit these patients and also, whether monitoring serum magnesium in recovering ARF patients is mandatory.
